# Sex-Biased Survival, Behavior Response, and Recovery Performance of *Pomacea canaliculata* Snails to Drought Stress and Rewatering Condition

**DOI:** 10.3390/biology12060768

**Published:** 2023-05-25

**Authors:** Chunxia Zhang, Zhaoji Shi, Yingtong Chen, Jing Guo, Jiaen Zhang, Zhong Qin

**Affiliations:** 1 Guangdong Engineering Technology Research Centre of Modern Eco-Agriculture and Circular Agriculture, South China Agricultural University, Guangzhou 510642, China; zzhangcx2023@163.com (C.Z.); shizhaojiyyy@gmail.com (Z.S.); yingtongchenchn@163.com (Y.C.); q_breeze@scau.edu.cn (Z.Q.); 2 College of Natural Resources and Environment, South China Agricultural University, Guangzhou 510642, China; jingaj@163.com; 3 Key Laboratory of Agro-Environment in the Tropics, Ministry of Agriculture and Rural Affairs, South China Agricultural University, Guangzhou 510642, China; 4 Henry Fok School of Biology and Agriculture, Shaoguan University, Shaoguan 512005, China

**Keywords:** *Pomacea canaliculata*, drought, rewatering, gender

## Abstract

**Simple Summary:**

In this study, we investigated the survival rate of *P. canaliculata* and explored the differential responses and mechanisms of female and male *P. canaliculata* under drought stress through a simulated drought experiment. Our findings revealed that female *P. canaliculata* produced a large number of eggs before burrowing into the soil when faced with drought condition. Furthermore, we observed that the feeding and activity of female *P. canaliculata* were more active than those of the males after rewatering following drought stress. Additionally, there were some gender differences in the antioxidant system of *P. canaliculata* after rewatering following drought stress. We expect that this research will be useful for a better understanding of how *P. canaliculata* tolerates and recovers from drought stress, which may aid in explaining the success of their invasions and predicting mollusk responses to climate change.

**Abstract:**

As the frequency of droughts increases with climate change, the tolerance of aquatic organisms to abiotic stressors will become critical determinants of survival. *Pomacea canaliculata* has become a widely distributed agricultural and environmental pest in southern China. To evaluate their tolerance and adaptation under the drought condition, the survival, feeding, behavior, and antioxidant system changes in female and male *P. canaliculata* were investigated during drought stress and rewatering process through an indoor simulation experiment. The results showed that female snails laid eggs before burrowing into the soil to ensure offspring reproduction. Female *P. canaliculata* had higher survival rates than males under drought stress, and their recovery ability of activity after rewatering was also superior to those of males. The antioxidant system of *P. canaliculata* showed obvious activation with gender differences after rewatering. Overall, the survival rate of female *P. canaliculata* was higher after drought stress, and the resilience ability of female snails after rewatering was stronger, including in their behavior, feeding, and antioxidant system recovery. The *P. canaliculata* tolerance to drought and the ability to recover quickly after drought may contribute to their long-term survival and facilitate continuous invasion.

## 1. Introduction

The apple snail, *Pomacea canaliculata* (Lamarck, 1822), originally from the Amazon River basin in South America, was introduced in the 1980s from Argentina into Asia for human food and the aquarium trade due to its rich protein content and high nutritional value [1,2]. It quickly spread to many countries in Southeast and East Asia, including many countries, such as Thailand, the Philippines, Laos, Cambodia, and Japan [3]. Since its introduction into China in 1981, *P. canaliculata* farming experienced a great upsurge [4]. However, the marketing failures have led to the abandonment of farm ponds used to rear these snails, allowing the snails to escape into rice fields and other wetland ecosystems [5]. They have rapidly spread to many provinces and cities south of the Yangtze River in China [6]. *P. canaliculata* is a notorious pest that causes serious damage to rice crop production by consuming large quantities of seedlings. Moreover, it serves as an intermediate host for *Angiostrongylus cantonensis*, a parasite that can cause eosinophilic meningitis in humans [7]. *P. canaliculata* has been listed as one of the 100 worst invaders in the world and is the only freshwater snail to appear on this list [8].

Global climate change has severely impacted freshwater ecosystems and is likely to match or surpass habitat destruction as the greatest cause of biodiversity loss in future [9]. It is expected to bring about extended and more severe droughts in many parts of the world [10]. Drought will directly alter species ranges and lead to the death of living individuals, while placing physiological pressures on many other surviving species, thereby reducing biodiversity in freshwater systems [11,12,13]. In addition, climate change will disproportionately affect many freshwater invertebrates, including *P. canaliculata*, by significantly affecting the temperature, quality, and quantity of water in aquatic ecosystems [14,15,16].

*P. canaliculata* is common in habitats that dry totally or partially, such as the paddy fields, alternating between wet and dry and the small ditches with scanty and variable discharge. Drought is a common abiotic stress experienced by apple snails [17], and it has been found that 17% of snails can survive up to 11 months under dry conditions in Japan [18]. Moreover, studies in southern China have also found that approximately 80% of snails can survive the winter in dry conditions [19]. It is predictable that the survival rate of overwintering *P. canaliculata* in the dry soil is closely correlated with the damage to the subsequent crop production and sustainability of aquatic ecosystems.

According to Yusa and Suzuki [20,21], sex ratios in juvenile *P. canaliculata* populations are not biased. However, natural populations of *P. canaliculata* usually exhibit a higher number of females than males, as reported by Halwart [22] and Tanaka et al. [23]. Female *P. canaliculata* have the ability to store sperm and can produce thousands of fertilized eggs from a single copulation event [24]. Moreover, a female *P. canaliculata* can lay hundreds of eggs at a time, which will usually be hatched within 15 d [25]. The hatching snails can reach sexual maturity in two to three months under favorable conditions [26]. The high proportion of females in *P. canaliculata* populations, coupled with their high fecundity, can lead to frequent population outbreaks that can cause significant damage to crop production and aquatic ecosystems. The reason for female dominance in *P. canaliculata* populations is not well understood, but it is possible that females possess greater adaptability to different habitats and stronger resistance to environmental stresses compared with males.

In this study, we conducted a simulated drought experiment by placing *P. canaliculata* in an indoor simulated paddy field soil environment and allowed them to drill into the soil, naturally. We tested the natural response of female and male *P. canaliculata* under drought stress, and recorded the survival rates after drought stress, as well as their behavioral dynamics after simulated drought followed by re-immersion. We also evaluated the changes in antioxidant enzymes in *P. canaliculata* under drought stress and re-immersion after drought stress. The objectives of this study were to investigate whether *P. canaliculata* can survive after drought and explore the differential responses and mechanisms of female and male *P. canaliculata* under drought stress by examining series of indicators. A more comprehensive understanding of how these snails tolerate and recover from drought stressors could help to explain their evolutionary and invasive success and to anticipate molluscan responses to climate change [27].

## 2. Materials and Methods

### 2.1. Experiment Materials

All of the tested *P. canaliculata* individuals were collected from irrigation ditches in a farm located at South China Agricultural University in Guangzhou (23°14′ N, 113°38′ E), Guangdong Province, China. A batch of eggs identified as *P. canaliculata* hatched in the irrigation ditches in advance, and a gauze net was installed at the inlet and outlet of the irrigation ditches to prevent apple snails from entering and escaping. Apple snails with a shell height of less than 25 mm and immature sex were selected during the collection to ensure that the selected snails had not experienced long-term drought before drought stress. Before the snails were used for the experiment, they were reared in an aquarium (70 cm long, 52 cm wide, 37 cm high) in a window-lit room with natural sunlight under temperatures of 24–30 °C, and they were fed with lettuce (*Lactuca sativa)* and duckweed (*Lemna minor)*. Female and male apple snails with a shell height of more than 30 mm (mature individuals) were selected for the experiment, based on exhibiting normal behavior and appearance. We first identified their sex based on operculum morphology, because the opercula at a particular site of males become convex when they tend to mature, which is different from the females whose opercula remain concave after sexual maturity [28]. Additionally, we further examined a visible albumin gland through the shell (to identify mature females) or testes (to identify mature males).

### 2.2. Experiment Procedure

One hundred and eighty tested female snails and the same-quantity tested male snails were assigned to each of the three treatments: control (aquatic condition) and two drought treatments, with drought durations of 60 and 120 d, respectively. Six replicates were performed for each of three treatments. Three treatments were placed in an outdoor balcony to ensure that they were as close as possible to the environmental field temperatures. Ten snails were released into each bucket (24 cm diameter and 21 cm high) at the beginning of the experiment. Each bucket was filled with an 8 cm layer of soil at the bottom and a 10 cm layer of water on top of the soil, and the buckets were covered with mesh to prevent the snails from escaping.

After 1 d adaptation, a siphon tube was used to slowly remove water at high water level, while a pipette and an ear syringe were used to meticulously extract water at low water level. This controlled the water level to decrease to the soil surface without any noticeable moisture in the two drought treatments. The water was slowly removed without losing soil, and the soil was allowed to dry naturally. When the remaining water level was lower than half of the apple snail’s shell height, the apple snails began to drill into the soil naturally, and the experiment began to record the drilling of female and male apple snails every hour. Subsequent experiments were conducted on the laboratory balcony, simulating natural soil drying without additional water input throughout the process.

Many eggs were found within the first day of lowering the water level, and all egg masses were carefully removed to a Petri dish, respectively. Eight milliliter pure aerated water was placed on the bottom cover of the petri dish, and then the top was covered with egg mass to ensure internal humidity and air circulation. Next, the dish was placed in an artificial climatic chamber with a 14/10 h (light/dark) photoperiod at 25 ± 2 °C. Hatchings were checked daily, and the incubation periods of these egg masses were calculated from the day when they were laid to the day of the first hatching [29]. In order to determine hatching success, the number of hatchlings in each egg mass was counted, and the rest of the non-incubated eggs were counted after they were dispersed in 2% sodium hydroxide solution for 1.5 h [30].

After arriving at the experimental processing time (drought duration of 60 and 120 days), 5 L aerated tap water was added to the buckets in the dry condition to bring the water level of 8 cm above the top of the soil. Survival status was confirmed when the snails were able to resume normal behavior after re-entering an aquatic condition. The behaviour of the snails was observed at 0.5, 0.75, 1, 1.5, 2, 3, 4, 5, and 6 h after rewatering. The individual behaviour of the snails was classified into six states of activity: feeding, crawling, foot deployment, first tentacle extension, aperture agape, and closed operculum. At the same time, the surviving snails were fed with pre-weighed lettuce, and their intake was assessed by measuring the wet weight of the surplus lettuce after 24 h.

Liver tissue (0.2 g) was sampled from eight randomly chosen snails (four of each female and male) per time at control (aquatic condition), under 120 d drought, and then 24 h after rewatering treatment (recovery aquatic condition was 24 h after 120 d of drought treatment). The liver was carefully removed with a sterile scalpel and washed with 0.9% NaCl before being dissected, and then it was drained on filter paper and stored at 80 °C in an ultra-low temperature refrigerator for enzymatic activity detection.

**Figure 1:**
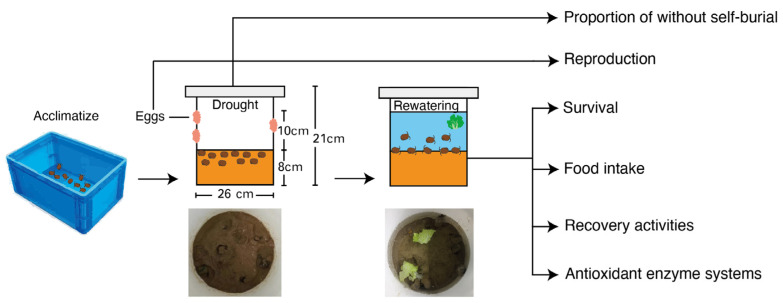
Experimental procedures to estimate survival, behavior response, and recovery performance of *P. canaliculata* snails to the drought stress and rewatering condition.

The activity of superoxide dismutase (SOD), catalase (CAT), total antioxidant capacity (T-AOC), glutathione S-transferase (GST), reduced glutathione (GSH), and malonaldehyde (MDA) were tested according to the operation manual of SOD (Product Code A001), CAT (Product Code A007), T-AOC (Product Code A015), GST (Product Code A004), GSH (Product Code A006), and MDA (Product Code A003) from Nanjing Jiancheng Company and the methods are described in the manufacturer’s operation manual (Figure 1).

### 2.3. Data Analysis

Independent sample t-tests were used to analyze the differences between males and females in self-burial, reproduction, survival, and food intake. Two-way analysis of variance was used to analyze the effects of sex and drought treatments on the antioxidant system of *P. canaliculata.* All analyses were performed using SPSS 22.0 (IBM Corp., Armonk, NY, USA). All graphs were produced using Origin 9.1 (Origin Lab, Northampton, MA, USA).

## 3. Results

**Figure 2:**
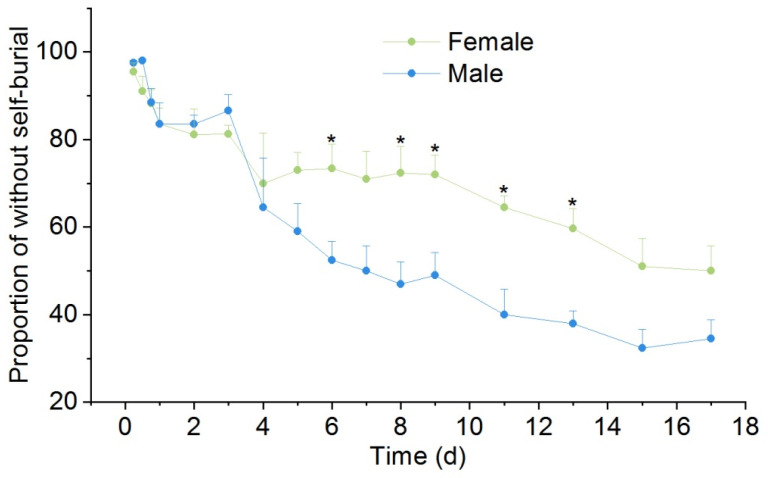
Effects of drought stress on the non-self-burial of *P. canaliculata*. The asterisks indicate significant differences in the proportion of without self-burial between female and male. * *p* < 0.05. Values are the mean ± SE.

Both female and male *P. canaliculata* started to self-bury when the water level was less than half of the shell. There was no significant difference in the non-self-burial proportion between female and male snails within the first 4 h, but the non-self-burial proportion in female snails was significantly higher than that in male snails at the 6, 8, 9, 11, and 13 h (*p* < 0.05), and the difference reached the maximum at the 11th h: the proportions of non-self-buried snails in female and male snails were 64.5% and 40%, respectively (Figure 2, Table 1).

**Figure 3:**
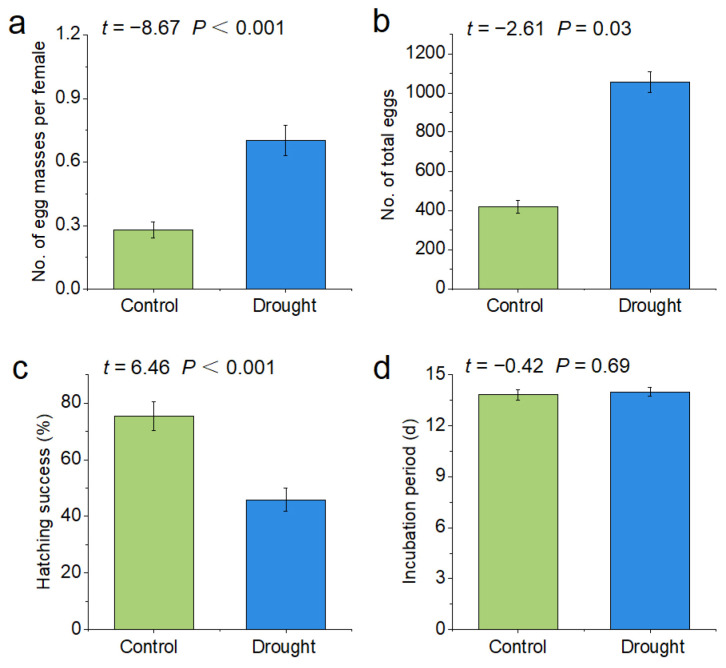
Effects of drought stress on the reproduction of *P. canaliculata*. The asterisks indicate significant differences in (**a**–**d**) ((**a**): no. of egg masses per female; (**b**): no. of total eggs; (**c**): hatching success; (**d**): incubation period) between the control and drought groups. Values are the mean ± SE.

Drought significantly increased the number of egg masses per female *P. canaliculata* (control: 0.28, drought: 0.7, *p* < 0.001) and the number of total eggs (control: 418.8, drought: 1056.6, *p* < 0.001), but significantly decreased the hatching rate (control: 75.39%, drought: 45.79%, *p* < 0.001) of *P. canaliculata*. The incubation period (control: 14 d, drought: 13.83 d, *p* > 0.05) did not change significantly under the drought treatment (Figure 3).

**Figure 4:**
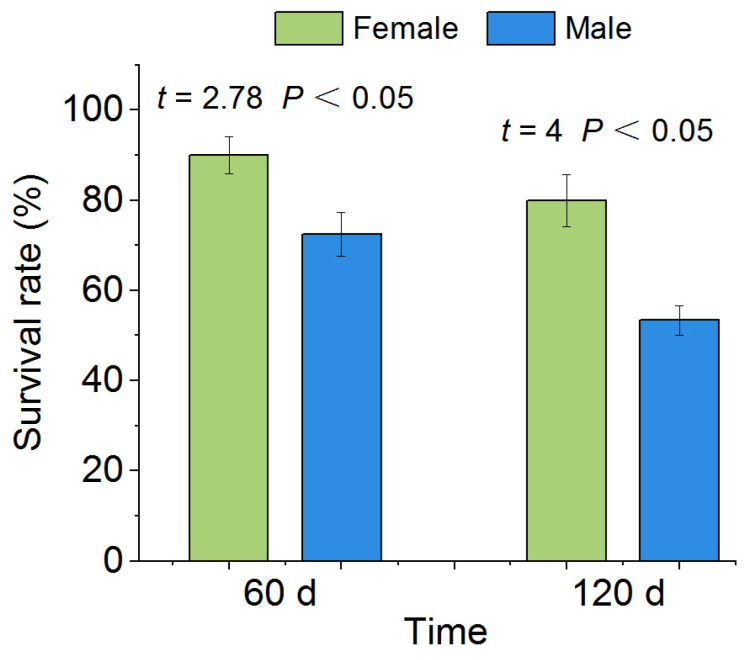
Survival rate after drought in *P. canaliculata*. The asterisks indicate that the survival rate of *P. canaliculata* was significantly different between females and males. Values are the means ± SE.

The average survival rates of female and male *P. canaliculata* after 60 d of drought were 90% and 72.5%, respectively, with a significant difference between female and male snails (*p* < 0.05, Figure 4). After 120 d of drought, the survival rates of both female and male *P. canaliculata* decreased compared with 60 d of drought, but the difference between female and male snails remained significant (female: 80%, male: 53.33%,* *p* < 0.05; Figure 4).

**Figure 5:**
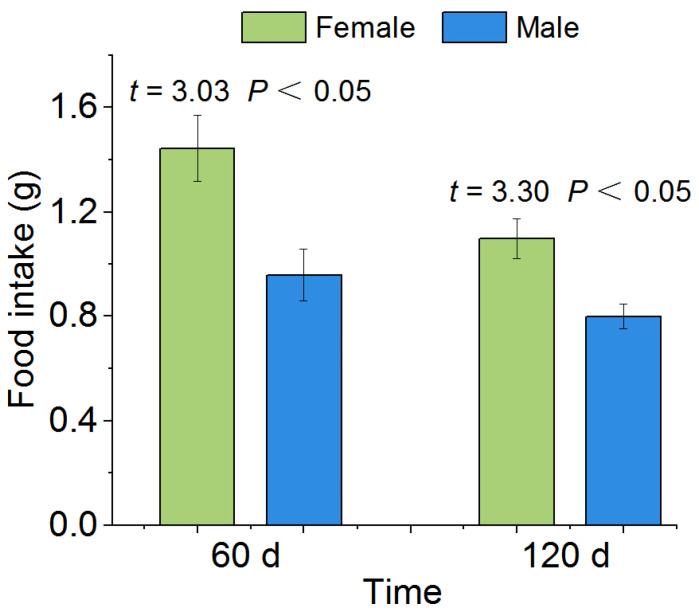
Effects of rewatering after the 60 d and 120 d drought stress on the food intake of single *P. canaliculata*. The asterisks indicate that the food intake of *P. canaliculata* was significantly different between females and males. Values are the mean ± SE.

When rewatered after 60 d drought, the feeding ability of *P. canaliculata* recovered (Figure 5). The results showed that the average 24 h food intake of female and male *P. canaliculata* was 1.44 g and 0.96 g, respectively, with a significant difference between female and male snails (*p* < 0.05, Figure 5). When rewatered after 120 d drought, the 24 h food intake of both female and male *P. canaliculata* was lower than that of the 60 d treatment, but there was still a significant difference between the female and male snails (female: 1.09 g, male: 0.79 g, *p* < 0.05; Figure 5).

**Figure 6:**
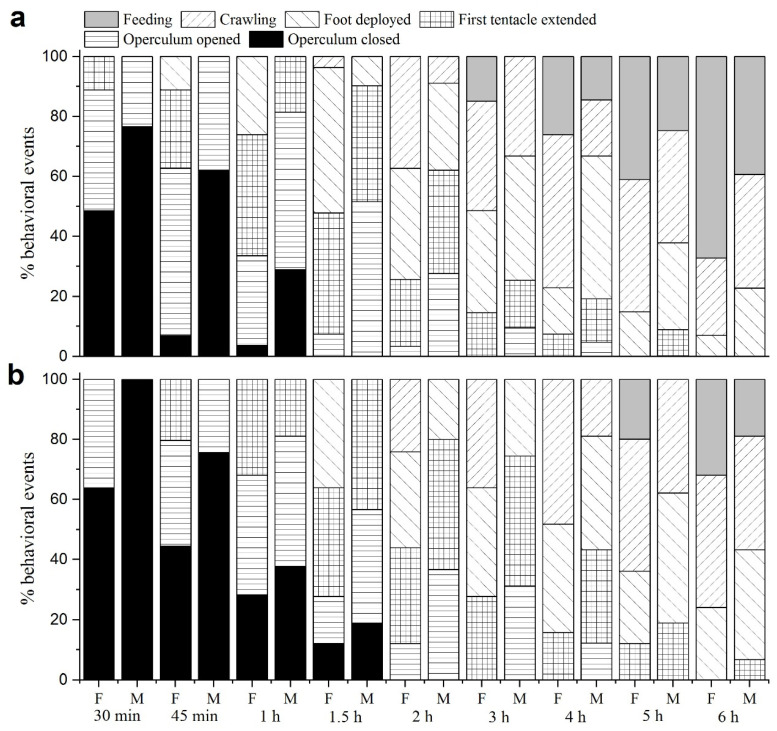
Activities observed for surviving snails during the first 6h after rewatering after the 60 d- or 120 d-drought ((**a**): drought duration of 60 d; (**b**): drought duration of 120 d). At each time point, the percentage of snails engaged in each activity is shown.

Figure 6a depicts the activity patterns of female and male *P. canaliculata* after being rewatered following 60 d drought. After 30 min, 40.28% of females began to open their operculum, and 11.20% even extended their tentacles. Males also showed signs of activity, with 23.41% starting to open their operculum. After 3 h, 14.91% of females began to feed, while 14.49% of male snails began to feed after 4 h. After 6 h, 67.22% of female snails and 39.29% of male snails were found at feeding status.

Figure 6b corresponds to the activity patterns of *P. canaliculata* after being rewatered following 120 d drought. After 30 min, 63.89% of females and all males had a sealed operculum. After 45 min, 20.37% of females first extended their tentacles, and 35.19% of females and 24.44% males opened their operculum. Meanwhile, 44.44% of females and 75.56% of males retained a closed operculum. After 2 h, all living snails showed signs of activity. The first instance of feeding was observed in females (19.91%) after 5 h, while males started feeding at least 6 h later. After 6 h, 31.94% of females and 18.89% of males kept feeding.

Through two-way analysis of variance, we found that drought stress significantly affected the activities of SOD (*p* < 0.001), MDA (*p* < 0.001), CAT (*p* < 0.001), T-AOC (*p* < 0.001), GST (*p* < 0.001), and GSH (*p* < 0.001) of *P. canaliculata*, while the snail sex significantly influenced the activities of SOD (*p* < 0.01), CAT, GST (*p* < 0.05), and GSH (*p* < 0.05) of *P. canaliculata*, and the interaction between the drought treatment and the snail sex significantly affected the GST activity (*p* < 0.05, Table 2).

**Figure 7:**
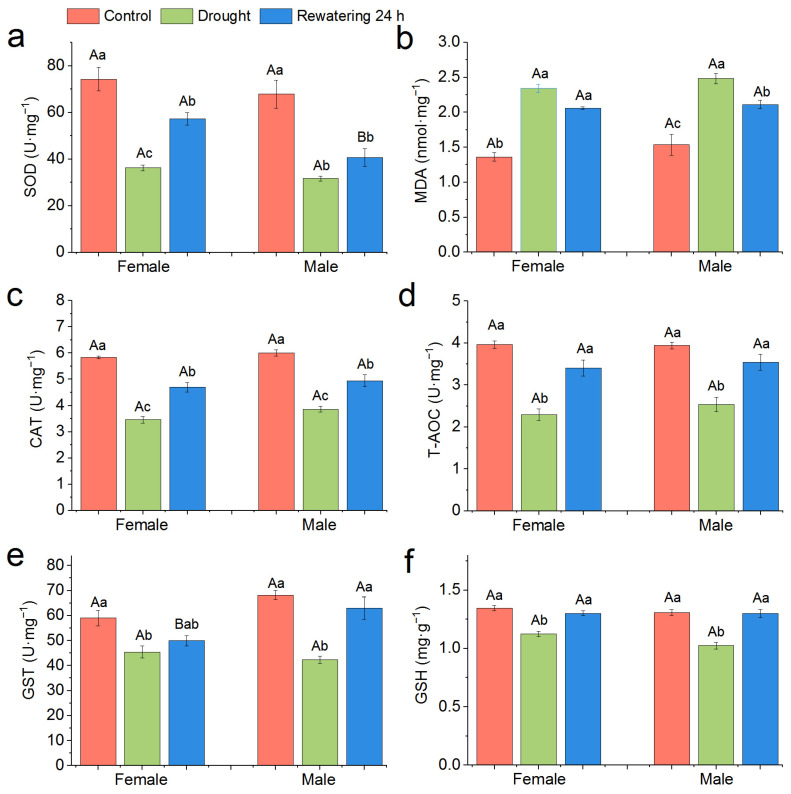
Effect of 120-d drought stress and rewatering treatment on antioxidant enzyme systems of *P. canaliculata*. (**a**): SOD; (**b**): MDA; (**c**): CAT; (**d**): T-AOC; (**e**): GST; (**f**): GSH. Different capital letters indicate significant differences between males and females in the same treatments; different lowercase letters indicate significant differences between control and drought stress and rewatering treatment in the same sex. Values are the means ± SE.

Specifically, the drought and then 24 h rewatering treatment significantly decreased SOD activity of both female and male snails, and female snails have higher SOD activity at 24 h after rewatering compared with male snails (Figure 7a). On the contrary, the drought and then 24 h rewatering treatment significantly increased MDA content for both female and male snails (Figure 6b). The CAT activity of both female and male snails significantly decreased after the drought stress and increased significantly after 24 h rewatering treatment, but it was still significantly lower than the control (Figure 7c). The T-AOC activity of both female and male snails significantly decreased under the drought stress, but it could reach the control level after 24 h rewatering treatment (Figure 7d). Drought significantly decreased the GST content of both female and male snails, and, at 24 h after rewatering, it did not significantly increase the GST content of female snails, but it significantly increased the GST content of male snails (Figure 7e). The GSH content of both female and male snails significantly decreased after drought but recovered to control level after 24 h rewatering (Figure 7f).

## 4. Discussion

Our results provide experimental evidence that both female and male *P. canaliculata* were able to survive extended periods of drying conditions that stimulated their natural burrowing behavior and allowed them to aestivate. After 120 d drought stress, 80% of female snails and 53.34% of males remained viable, which supports the snails’ strong drought tolerance. Darby et al. recorded *P. paludosa* had a 70% survival rate after 84 d in a comparable simulated outdoor dry down event [31]. In another study [19], more females (44.7%) survived than males (32.1%) in the drying conditions created by full burial after 47 d. Moreover, they could survive in dry conditions for up to 13 months, and they were buried in wet mud for up to 29 months. Differences in exposure method, snail size, and species may contribute to the significant difference in survival rates between previous studies and our study.

In addition, the survival rate of females was significantly higher than that of males under drought treatments, indicating that *P. canaliculata* females exhibited greater viability than males in terms of drought tolerance. Sex-biased drought tolerance has also been found in other animals. Male elephants (*Loxodonta africana*) are more sensitive to drought than female elephants [32]. Previous research has shown that *Pomacea sp*. females exhibited greater viability than males in terms of desiccation tolerance [33]. Other aspects of *P. canaliculata* have also shown sexual differences. In addition to a longer lifespan [34], females also exhibit faster growth rates than males [26]. When confronted with predation risks, females have been shown to respond more strongly to alert signals from conspecifics, resulting in a higher survival rate than males in interactions with natural predators [35]. In terms of susceptibility to high temperatures and fasting, males seem to be more vulnerable than females [36,37]. These dissimilarities between female and male *Pomacea* sp. might be attributed to a female survival strategy that prioritizes preserving reserves and nutritional condition, while males tend to deplete their energy reserves to increase their reproductive success [37].

The high survival rate of *P. canaliculata* that we observed after 60 and 120 d of dry conditions is best explained by their diverse suite of behavioral and physiological adaptations for tolerating abiotic stressors. *P. canaliculata* with a dual respiratory system consisting of a gill and a lung, has a variety of traits, such as having a flexible operculum, burrowing behavior, and living both in water and on dryland, which all probably contribute to the survival [27,38]. In this study, it was observed that the recovery was not immediate, and the transition out of dry conditions progressed gradually. Surviving *P. canaliculata* also survived and resumed normal activities of their return to water condition. From opening the aperture to first extending a tentacle and beginning movement, *P. canaliculata* became active, then deployed their foot, crawled, and resumed feeding. Giraud-Billoud et al. recorded similar behavioral recovery of *P. canaliculata* individuals [39]. Glasheen et al. [40] further observed that snails of the *Pomacea* species resume mating and can reproduce successfully after extended exposure to drying conditions. The recovery process of activity after rewatering following 60 and 120 d of drought was found to be better in female *P. canaliculata* than in males. After 60 d drought, females began feeding at 3 h after rewatering, while males needed 4 h to recover feeding. Females and males resumed feeding at 5 and 6 h after 120 d drought, respectively. Additionally, after 24 h, the feeding amount of female snails was significantly higher than that of males. This suggests that females have better resilience to recover from drought than males, and the tolerance to drought and high population resilience after rewatering may partially account for the success of *P. canaliculata* as expanding global invaders [41,42].

This study reports for the natural response of female and male *P. canaliculata* in drying conditions found that far fewer females than males buried themselves in sediment during the first few hours of drought. Experimental observations suggest that females delayed burrowing in order to lay eggs, and most females began laying eggs at midnight, 17 h after the start of the experiment, while males continued to move or remain stationary. However, by the 24th h of the experiment, most snails of both sexes had buried themselves into the soil. Compared with controls, *P. canaliculata* under the dry conditions significantly increased spawning quantity at the first day of the experiment. Guo et al. found that, at the beginning of the experiment, under predation risk, *P. canaliculata* laid eggs faster and produced more eggs [43]. Another freshwater snail, *Biomphalaria glabrata*, also showed an increase in egg production shortly after exposure to a trematode parasite without parasitization [44]. Increased egg production may be a strategy for snails to maximize reproductive success when facing serious threats. The phenomenon of snails laying a large number of eggs under unfavorable conditions in the natural environment is beneficial for ensuring offspring and population reproduction. This indicates that, in addition to preserving themselves, snails will also allocate some of their energy to population reproduction when they encounter sudden changes in their environment. Invasive *P. canaliculata* produce large numbers of eggs with high hatching success [45,46]. Even if only a small number of *P. canaliculata* survive, the population would continue to grow and could cause more harm [47].

In the antioxidant system of animals, SOD, CAT, T-AOC, GST, and GSH have the function of scavenging reactive oxygen species (ROS), playing an important role in enhancing the defense and immune function of phagocytes [48,49]. However, antioxidant enzyme activities did not increase during drought, which may be because of the general inhibition of protein synthesis due to hypometabolism of the *P. canaliculata* during the estivation under drought [49]. Correspondingly, an increase in MDA content was observed. Lipid peroxidation is considered the result of ROS oxidizing lipids, and MDA is considered one of the final decomposition products of lipid peroxidation, reflecting the degree of oxidative damage to cell membranes indirectly [50,51]. It is worth noting that, after 24 h rewatering, the antioxidant enzyme activities of female and male *P. canaliculata* both increased, and their corresponding MDA content also decreased. In a previous study, an increase in TBARS (an index of oxidative stress-induced lipid peroxidation damage) was observed in the soft part of the snail during estivation, but it decreased to the level of active control after awakening [39], which is in line with the change of MDA in our study. Moreover, there were some gender differences in the antioxidant system of *P. canaliculata* after the rewatering under drought stress. For example, after 24 h rewatering, females exhibited stronger resilience of SOD activity while demonstrated weaker resilience of GST activity compared with males. This may indicate differences in the mobilization patterns of antioxidant enzymes in female and male *P. canaliculata.*

Paddy fields are the primary habitat of *P. canaliculata* in invaded areas of Asia, which experience alternating submerged and dry conditions during rice cultivation and fallow periods. The ability of *P. canaliculata* to withstand drought in this complex and changing environment facilitated its colonization. Consequently, *P. canaliculata* populations have been able to establish themselves in many regions and countries in Asia [6,52]. Our findings provide evidence of one of the key traits that contribute to the colonization of *P. canaliculata* in paddy fields.

## 5. Conclusions

The present study found that female *P. canaliculata* produced a large number of eggs before burrowing into the soil when facing with drought condition and had a higher survival rate than males under drought stress, and their feeding and activity after rewatering are also more active than those of males. The antioxidant system of *P. canaliculata* showed significant activation after 120 d drought and a gender difference. Drought tolerance and the ability to recover quickly after drought may benefit *P. canaliculata* invasion, which would cause more serious damage on crop production and the sustainability of aquatic ecosystems.

## Figures and Tables

**Table 1:** Independent sample *t* test for the proportion of non-self-burials in *P. canaliculata*.

Hour	*t*	*p*
0.25	0.9828896	0.416498475
0.5	1.9836509	0.179002723
0.75	0.044475	0.966657533
1	0	1
2	0.3817977	0.733035165
3	1.248705	0.298132643
4	−0.341041	0.750244106
5	−1.864201	0.148449309
6	−3.00944	0.042325305
7	−2.450582	0.07097177
8	−3.193243	0.034211695
9	−3.402145	0.02852897
11	−3.869761	0.034984118
13	−3.982427	0.023027933
15	−2.423914	0.08097939
17	−2.152339	0.103095003

**Table 2:** Two-way analysis of variance of drought on antioxidant systems in *P. canaliculata*.

Variable	Drought			Gender			Drought * Gender		
	MeanSquare	F	*p*	MeanSquare	F	*p*	MeanSquare	F	*p*
SOD	3310	62.472	<0.001	667	12.588	<0.01	114	2.142	0.13847
MDA	2.2349	83.396	<0.001	0.109	4.065	0.0546	0.0089	0.332	0.7205
CAT	12.538	103.99	<0.001	0.53	4.396	0.0463	0.048	0.401	0.674
T-AOC	6.341	46.458	<0.001	0.169	1.24	0.276	0.041	0.304	0.741
GST	997	27.669	<0.001	240.1	6.663	0.0161	188.4	5.228	0.0127
GSH	0.20584	55.735	<0.001	0.01669	4.518	0.0436	0.00769	2.082	0.1458

## Data Availability

The data for the analysis in this study are available upon request from the corresponding author.
